# Gender-Related Impact of Sclerostin Antibody on Bone in the Osteogenesis Imperfecta Mouse

**DOI:** 10.3389/fgene.2021.705505

**Published:** 2021-08-10

**Authors:** Mickaël Cardinal, Antoine Chretien, Thomas Roels, Sébastien Lafont, Michael S. Ominsky, Jean-Pierre Devogelaer, Daniel H. Manicourt, Catherine Behets

**Affiliations:** ^1^Pole of Morphology, Institut de Recherche Expérimentale et Clinique, UCLouvain, Brussels, Belgium; ^2^Radius Inc., Waltham, MA, United States; ^3^Amgen Inc., Thousand Oaks, CA, United States; ^4^Pole of Rheumatic Pathologies, Institut de Recherche Expérimentale et Clinique, UCLouvain, Brussels, Belgium

**Keywords:** osteogenesis imperfecta, oim/oim, sclerostin antibody, fracture, bone quality, biomechanical strength, gender

## Abstract

Osteogenesis imperfecta (OI), which is most often due to a collagen type 1 gene mutation, is characterized by low bone density and bone fragility. In OI patients, gender-related differences were reported, but data in the literature are not convergent. We previously observed that sclerostin antibody (Scl-Ab), which stimulates osteoblast Wnt pathway via sclerostin inactivation, improved spine and long-bone parameters and biomechanical strength in female oim/oim mice, a validated model of human type 3 OI. Here, we wanted to highlight the effect of Scl-Ab on male oim/oim bones in order to identify a possible distinct therapeutic effect from that observed in females. According to the same protocol as our previous study with female mice, male wild-type (Wt) and oim/oim mice received vehicle or Scl-Ab from 5 to 14 weeks of age. Clinimetric and quantitative bone parameters were studied using X-rays, peripheral quantitative computed tomography, microradiography, and dynamic histomorphometry and compared to those of females. Contrary to Wt mice, male oim/oim had significantly lower weight, snout–sacrum length, and bone mineral content than females at 5 weeks. No significant difference in these clinimetric parameters was observed at 14 weeks, whereas male oim showed significantly more long-bone fractures than females. Scl-Ab improved bone mineral density and bone volume/total volume ratio (BV/TV) of vertebral body in Wt and oim/oim, without significant difference between male and female at 14 weeks. Male vehicle oim/oim had a significantly lower cortical thickness (Ct.Th) and BV/TV of tibial diaphysis than female and showed a higher number of fractures at 14 weeks. Scl-Ab increased midshaft periosteal apposition rate in such a way that tibial Ct.Th of male oim/oim was not significantly different from the female one at 14 weeks. The number of fractures was lower in male than female oim/oim after 14 weeks of Scl-Ab treatment, but this difference was not significant. Nevertheless, Scl-Ab–treated oim/oim male and female mice remained smaller than the Wt ones. In conclusion, our results highlighted differences between male and female oim/oim at 4 and 14 weeks of age, as well as some male-specific response of cortical bone to Scl-Ab. These gender-related particularities of oim/oim should be considered when testing experimental treatments.

## Introduction

Low bone density and bone fragility are the hallmarks of osteogenesis imperfecta (OI), a heterogeneous group of heritable bone disorders often caused by a mutation affecting one of the two genes COL1A1 and COL1A2 that encode collagen type I chains (Rauch and Glorieux, 2004; van Dijk et al., 2011). OI is the most frequent bone dysplasia (8 cases per 100,000 live births) (Stevenson et al., 2012) and a source of significant disability (Engelbert et al., 2004).

The phenotype of OI is variable and depends on the type and position of the causative mutation (Rauch and Glorieux, 2004; Tournis and Dede, 2018). Prognosis and therapeutic evaluation are based on an increasingly complex classification associating causative genes and phenotypes. Briefly, it distinguishes phenotypes with mild to moderate severity (types 1, 4, 5), a progressively deforming phenotype (type 3), and a perinatally lethal phenotype (type 2) (Van Dijk and Sillence, 2014). Type 3 is the most severe form in children surviving the perinatal period: the symptoms are related to low bone mass and consist in high bone fragility, multiple bone fractures from minimal traumas, and skeletal deformities affecting axial or appendicular bones (Wekre et al., 2011). The fragility resulting from the poor matrix quality is worsened by sluggish periosteal and trabecular bone formation. Long bones present smaller external diameter and cortical thickness (Ct.Th) than those of normal subjects. Fragility fractures of vertebral bodies cause spine curvature disorders (McAllion and Paterson, 1996).

In addition, defects in type I collagen molecules provide an abnormal scaffold for mineral deposition, which decreases bone plasticity and favors the formation of microcracks. These defects are also responsible for a variety of extraskeletal symptoms that can be found, such as hyperlaxity, dentinogenesis imperfecta, blue sclera, and frequent loss of hearing (Khandanpour et al., 2012).

In a Norwegian database, fractures were found more frequent in men than women with OI (aged 44 ± 12 years), whatever the type of OI (Wekre et al., 2011). Furthermore, girls with OI seem to have a higher bone mineral density (BMD) than boys at the end of adolescence [aged 17.4 and 17.7 years, respectively (Kok et al., 2013)]. However, a former study had shown that the fracture rate was higher in OI women than in OI men in childhood, in adolescence, and even more during the menopause, whereas bones were frailer in adult men (Paterson et al., 1984). Such discrepancy in the skeletal data between male and female was also reported in oim/oim mice (Vanleene et al., 2012; Yao et al., 2013; Boskey et al., 2015).

Recently, we demonstrated that sclerostin antibody (Scl-Ab) improves long-bone (Cardinal et al., 2019) and vertebral bone (Cardinal et al., 2020) parameters in female oim/oim. Indeed, this treatment enhanced the mechanical properties of long bones while increasing osteoblastic apposition, BMD, and cortical bone geometry. These changes were associated with a significant reduction in the number of long-bone fractures. In addition, the therapy also reduced the fracture rates in the axial skeleton by enhancing the cortical shell of vertebrae, improving vertebral trabecular bone mass, microarchitecture, and resistance to compressive forces. Consequently, we wanted to know if the Scl-Ab could improve bone formation in long bone (here tibia) and in vertebral bodies of male oim/oim and thereby to highlight a possible distinct therapeutic effect between males and females.

## Materials and Methods

### Animals and Treatment

Five-week-old male oim/oim (OI) (strain B6C3Fe a/a-Col1a2oim/J) and wild-type (Wt) mice (strain B6C3Fe-a/a + / + ; SN 1815) (Charles River Laboratories, 69592 L’Arbresle, France) received intraperitoneal injections of either Scl-Ab (50 mg/kg) (Amgen, Thousand Oaks, CA, United States) or vehicle (Veh.) once a week for 9 weeks. As OI mice show marked bone fragility, animals were sedated with volatile anesthetic sevoflurane (Cesarovic et al., 2010) to facilitate gentle handling. The mice were weighed at 5 weeks of age and before each injection. To allow dynamic bone histomorphometry, calcein green (Sigma-Aldrich, St. Louis, MO, United States; 10 mg/kg) was injected intraperitoneally at days 1, 21, 42, and 63 of treatment. The mice were euthanized using a volatile anesthetic Sevoflurane overdose (SEVOrane, Quick Fill, Abbott, Belgium) at 14 weeks of age. The right tibia and the spine were immediately dissected and stored in 70% methanol. All experimental procedures were approved by the local ethics committee for animal care of the Université catholique de Louvain (ethical committee authorization no. 2014/UCL/MD/021), and the animals were housed according to the Belgian Federal Public Health regulations. It is also important to specify that this experiment was conducted at the same time and in the same conditions as that with female mice used for the present comparison (Cardinal et al., 2019, 2020).

### Fracture Count

A mammography system allowed obtaining high-resolution anteroposterior and mediolateral digital radiographs. On completion of the study, we radiographed all mice of both OI groups after sacrifice (OI Veh. *n* = 15 and OI Scl-Ab *n* = 13). Two independent observers blinded to the group assignment counted the fractures in the femurs, tibias, humerus, and forearms. Solution of continuity, callus formation, obvious bone deformity, or compression were defined as fracture. The tibias with fracture were excluded from further analyses.

### Dual-Energy X-Ray Absorptiometry

*In vivo* whole-body bone mineral content (BMC) was assessed by dual-energy X-ray absorptiometry (QDR Discovery 4500 A, Hologic, Belford, MA, United States) at 4 and 14 weeks of age. Body size was evaluated by measuring the snout–sacrum length.

### Bone Geometry

Each right tibia (*n* = 10/group) was radiographed with soft X-rays (14 kV, 15 mA). The total length was measured by two independent observers on digitized radiographs with ImageJ program (ImageJ 1.43u, W. Rasband, National Institutes of Health, United States).

### Peripheral Quantitative Computed Tomography

The tibias (*n* = 10/group) and spines (*n* = 10/group) of OI and Wt mice were scanned *ex vivo* with a peripheral quantitative computed tomography (pQCT) Research SA + (Stratec, Birkenfeld, Germany). Images were obtained at a slice thickness of 150 μm, and the voxel size was 0.07 mm. The threshold was 570 mg/cm^3^ for cortical bone and 280 mg/cm^3^ for cancellous bone (Cardinal et al., 2019). The tibias were scanned in the transversal plane through midshaft. The spines were scanned in the sagittal plane. Slices were analyzed with the XCT540 software of the pQCT in order to obtain BMD, cross-sectional area (CSA), and length (rostrocaudal height excluding the intervertebral discs), as well as polar stress–strain index (SSI), a surrogate measure of torsional bone strength. All the parameters were measured in an average of three adjacent slices of each bone or spine.

### Microradiography

Tibias (*n* = 8/group) and spines (*n* = 10/group) were dehydrated with ascending grades of methanol, defatted in chloroform, and cleared in toluol, without any decalcification. After impregnation with methyl methacrylate (MMA) monomer at 5°C under vacuum for 48 h, a catalyst (anhydrous dibenzoyl peroxide) was added to polymerize MMA at 36°C for 4 to 7 days. After polymerization, the tibias were cut crosswise at the mid-diaphysis and the spines were cut into 150-μm-thick sagittal slices using a circular diamond saw (Leica SP1600, Nussloch, Germany). The sections were sanded manually on a ground-glass plate to obtain a thickness of 100 ± 1 μm (Cornelis et al., 2008). They were then placed on a fine-grain holographic emulsion (VRP-M, Slavich Geola, Vilnius, Lithuania) and microradiographed with a Machlett tube with anode of tungsten (Baltograph, Balteau, Liège, Belgium). The exposure time was 50 minutes at 14 kV and 13 mA. The films were revealed with SM-6 developer (Geola), fixed (Ilford 2000RT), and rinsed in tap water. The microradiographs were dried and digitized. The Ct.Th and relative bone volume (BV/TV) of tibias and the trabecular bone sectional area (TBS) and trabecular BV/total volume ratio (BV/TV) in vertebral body of T2 and L5 were measured with ImageJ.

### Dynamic Histomorphometry

We acquired fluorescence images of the undecalcified MMA sections through the tibia diaphysis (*n* = 6/group) with an Axio Scope.A1 microscope (Zeiss, Jena, Germany) in order to highlight calcein green labeling (excitation 485/20 nm, emission 540/25 nm) (Little et al., 2017). On these images, we delineated bone included between the first (day 1) and the last (day 63) labels of calcein at the periosteal and endosteal circumference by using ROI Manager of ImageJ program. Then, we measured the average thickness of bone between the labels with a ring model in order to compute the mineral apposition rate (MAR) and bone formation rate (BFR).

### Statistical Analysis

All quantitative data are expressed as mean ± SD in tables or mean + SEM in graphs. The data were analyzed using GraphPad InStat (version 3.10, GraphPad Software, Inc., San Diego, CA, United States). Parametric data were analyzed by Tukey–Kramer test with a simple analysis of variance (one-way ANOVA) for multiple comparisons. If the group variances were significantly heterogeneous (*p* < 0.05), a Kruskal–Wallis non-parametric one-way ANOVA test was used and followed by a Dunn test for *post hoc* multiple comparisons. A one-tailed Student *t* test was used to assess the differences between mice genders.

## Results

### Clinimetry

Male oim/oim mice treated with vehicle (OI Veh.) were significantly lighter and smaller than Wt mice treated with vehicle (Wt Veh.) at both 5 and 14 weeks of age (Table 1). Scl-Ab treatment did not change these differences between OI and Wt. The four groups gained weight (Wt Veh.: + 73%, Wt Scl-Ab: + 82%, OI Veh.: + 82.4%, OI Scl-Ab: + 81%) and grew (Wt Veh.: + 10.5%, Wt Scl-Ab: + 13%, OI Veh.: + 18%, OI Scl-Ab: + 16.5%) similarly during the treatment. Thus, treatment with Scl-Ab did not change weight and length growth in both genotypes.

**TABLE 1:** Weight, snout–sacrum length (S-S L), and whole-body bone mineral content (BMC) assessed with dual-energy X-ray absorptiometry in male wild-type (Wt) and oim/oim (OI) mice before and after 9-week treatment with either vehicle (Veh.) or Scl-Ab.

	Wt Veh.	Wt Scl-Ab	OI Veh.	OI Scl-Ab
	*n* = 12	*n* = 12	*n* = 14	*n* = 16
**Weight (g)**				
5 weeks	18.9 ± 4.4	18.5 ± 6.2	11.4 ± 2.6 a***	12.6 ± 3.1 c***
Gender difference (%)	−5.2	−10.8	−35.9***	−24.6**
14 weeks	32.8 ± 3.1	33.7 ± 3.9	20.8 ± 5.8 a***	22.8 ± 3 c***
Gender difference (%)	+ 23.1***	+ 20.4***	+ 7.6	+ 4.3
Gain	13.8 ± 3.3	15.2 ± 3.4	9.4 ± 5.1 a*	10.2 ± 3.1
**S-S L (cm)**				
5 weeks	8.6 ± 0.7	8.5 ± 0.5	6.9 ± 0.6 a***	7.3 ± 0.4 c***
Gender difference (%)	+ 4.6*	+ 2.3	−10.1***	−5.4**
14 weeks	9.5 ± 0.2	9.6 ± 0.3	8.2 ± 0.9 a***	8.5 ± 0.4 c***
Gender difference (%)	+ 5.2***	+ 4.1**	−1.2	−1.1
Gain	0.9 ± 0.7	1.1 ± 0.9	1.3 ± 0.7	1.2 ± 0.4
**BMC (mg)**				
5 weeks	446.6 ± 93.7	490 ± 12.2	222.1 ± 48 a***	252.5 ± 48.1 c***
Gender difference (%)	+ 3.7	+ 6.7	−26.9***	+ 24.7**
14 weeks	735 ± 87.8 c***	1018.3 ± 116.7 b***	415 ± 111.4 a***	526.8 ± 98.3 b*
Gender difference (%)	+ 17***	+ 16.7***	+ 9.3	−0.7
Gain	288.4 ± 82.5	528.3 ± 104.7 b***	192.9 ± 97.9	274.3 ± 79.8

The data from females used for gender differences (%) were obtained in the same experimental conditions, as described previously (Cardinal et al., 2020). Values are mean ± SD. a: OI Veh. versus Wt Veh., b: Scl-Ab versus Veh., c: OI Scl-Ab versus Wt Veh. *p < 0.05, **p < 0.01, ***p < 0.001.

Whole-body BMC was also significantly lower in OI Veh. than in Wt Veh. at the beginning (−101%), as well as at the end of the experiment (−77%). All groups increased their BMC during the 9 weeks of the experiment (Wt Veh.: + 64.5%, Wt Scl-Ab: + 102%, OI Veh.: + 86%, OI Scl-Ab: + 109%). Scl-Ab treatment significantly increased the BMC in Wt (+ 38.6%) and in OI (+ 27%) compared to vehicle counterparts. However, BMC of the OI Scl-Ab remained significantly lower than that of Wt Veh.

At 5 weeks of age, the weight, snout–sacrum length, and BMC of the male Wt mice did not differ from those of the female counterparts (Table 1). The male OIs were significantly lighter (−35.9%) and smaller (−10.1%) and had lower BMC (−26.9%) than female OI. After 9 weeks of treatment, in both male Wt groups, all these parameters were significantly greater than in female Wt (Table 1). In OI mice, whatever the treatment, no difference was observed in weight, snout–sacrum length, and BMC between males and females. Scl-Ab did not appear to impact these parameters in both males and females.

### Fracture Count

No fracture was observed in male Wt mice. At the end of the experiment, the fractures were significantly more numerous (+29%) in male than female OI Veh (Table 2). Scl-Ab treatment decreased significantly the fracture number in both males (−72.1%) and females (−51.1%). This reduction in the number of fractures was greater in males than in females. Indeed, OI Scl-Ab males tended to have fewer fractures (−23.3%) than their female counterparts.

**TABLE 2:** Mean number of long-bone fractures per mouse observed in male and female OI mice after 9-week treatment with vehicle (Veh.) or Scl-Ab.

	OI Veh.	OI Scl-Ab
Male	*n* = 15	*n* = 13
	4.13 ± 0.6 a***	1.15 ± 0.4
Female	*n* = 10	*n* = 8
	3.20 ± 0.5 b***	1.50 ± 0.6 a***

Values are mean ± SD. a = OI Veh. versus OI Scl-Ab; b = male versus female. ***p < 0.001.

### Vertebral Bone Analysis

**FIGURE 1:**
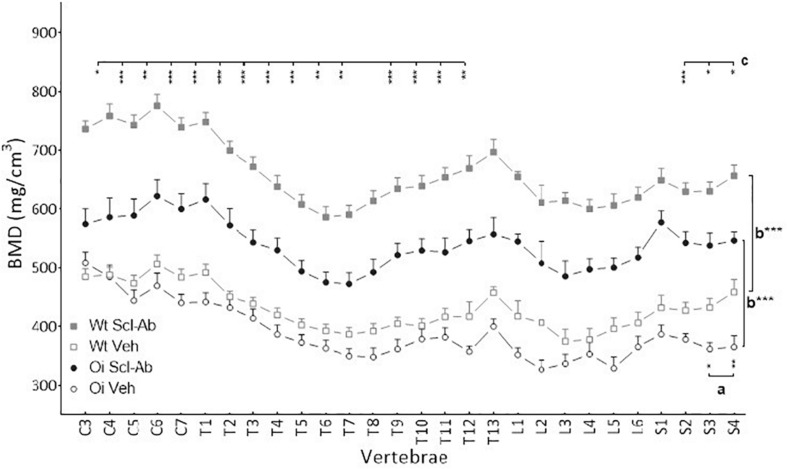
Vertebral body bone mineral density (BMD) from C3 to S4 assessed by sagittal pQCT in male wild-type (Wt) and oim/oim (OI) mice after 9-week treatment with either Scl-Ab or vehicle (Veh.). Results are expressed as mean + SEM; a = OI Veh. versus Wt Veh.; b = Scl-Ab versus Veh., c = OI Scl-Ab versus Wt Veh.; *p < 0.05, **p < 0.01, ***p < 0.001.

**FIGURE 2:**
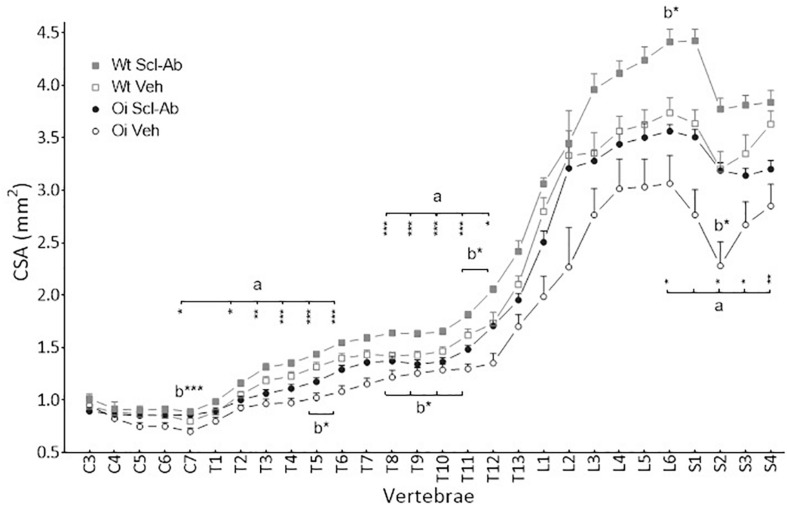
Vertebral body cross sectional area (CSA) from C3 to S4 assessed by sagittal pQCT in male wild-type (Wt) and oim/oim (OI) mice after 9-week treatment with either Scl-Ab or vehicle (Veh.). Results are expressed as mean + SEM; a = OI Veh. versus Wt Veh.; b = Scl-Ab versus Veh., *p < 0.05, **p < 0.01, ***p < 0.001.

**FIGURE 3:**
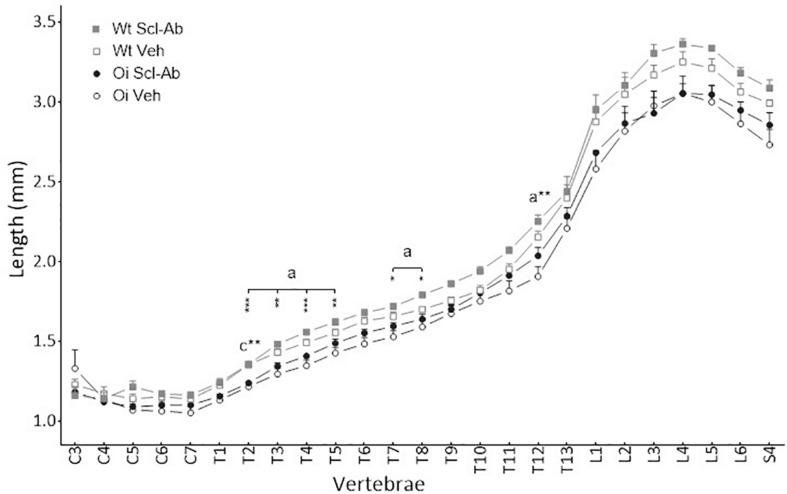
Vertebral body length from C3 to S4 assessed by sagittal pQCT in male wild-type (Wt) and oim/oim (OI) mice after 9-week treatment with either Scl-Ab or vehicle (Veh.). Results are expressed as mean + SEM; a = OI Veh. versus Wt Veh.; c = OI Scl-Ab versus Wt Veh.; *p < 0.05, **p < 0.01, ***p < 0.001.

At 14 weeks, BMD of male OI Veh. vertebral body was lower than that of Wt Veh., but this difference was significant only in S3–S4 vertebrae (Figure 1). Scl-Ab treatment significantly increased vertebral body BMD in OI and in Wt as compared with the Veh.-treated groups. After 9 weeks of treatment, BMD was even significantly greater in most cervical, thoracic, and sacral vertebral bodies of OI Scl-Ab than Wt Veh. ones. The CSA of vertebral bodies was lower in OI Veh. than in Wt Veh. mice, particularly in thoracic and sacral vertebrae in which the difference was significant (Figure 2). Scl-Ab tended to increase CSA in both Wt and OI vertebrae, but this increase was significant in only a few thoracic vertebrae. Despite shorter vertebral bodies in most spinal regions of OI Veh. than Wt Veh., only T2 to T5 and T7 to T8 vertebral bodies were significantly smaller in OI than Wt mice (Figure 3). The Scl-Ab treatment did not significantly change this parameter, so that no significant difference in length was observed between Wt Veh. and OI Scl-Ab, except in T2.

At 14 weeks, BMD of cervical and thoracic vertebrae was significantly lower in male than in female Wt Veh. (data not shown, see Supplementary Material Annex). The length of these vertebral segments was significantly higher in male than in female Wt Scl-Ab (+ 16.2% for C6). Thoracic vertebrae of male OI Veh. had also lower BMD than those of female counterparts (−14.8% for T8). We did not find any significant difference in cervical and thoracic vertebrae between male and female OI treated with Scl-Ab. CSA of lumbar and sacral vertebrae was significantly higher in male Wt Veh. and Wt Scl-Ab than female counterparts. In OI Scl-Ab, CSA of sacral vertebrae was higher in males than females.

**FIGURE 4:**
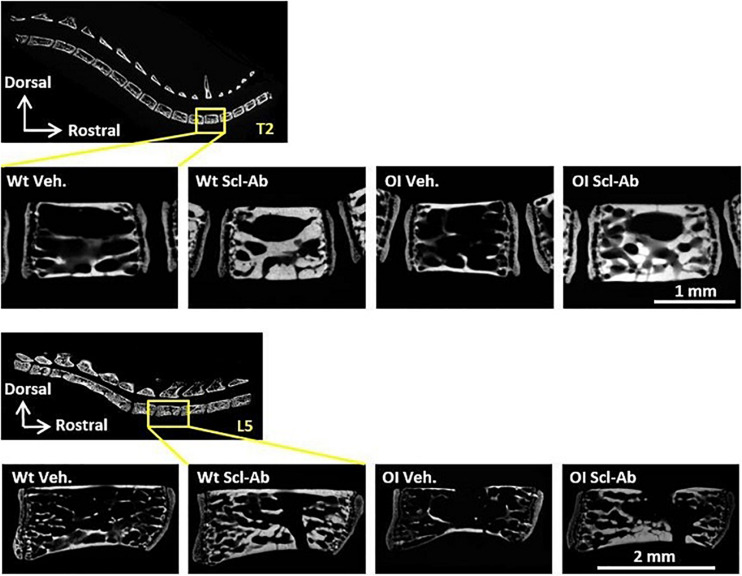
Microradiograph of sagittal undecalcified sections through T2 and L5 vertebral bodies of male Wt Veh., Wt Scl-Ab, OI Veh., and OI Scl-Ab mice.

At microradiographic analysis of undecalcified sagittal slices through the spine, the radiopacity, trabecular BV, and Ct.Th of vertebral bodies appeared lower in OI Veh. than Wt Veh. mice (Figure 4). Scl-Ab-therapy markedly improved these qualitative parameters in both OI and Wt mice. After 9-week therapy, vertebral bodies appeared similar in OI Scl-Ab and Wt Veh. mice. We did not detect fracture in cervical, thoracic, and lumbar vertebrae. On these microradiographs, we quantified trabecular BV fraction (BV/TV) and sectional area (TBS) of T2 and L5 vertebral bodies. No significant difference in BV/TV was observed between male Wt Veh. and OI Veh. (Table 3). Scl-Ab significantly enhanced BV/TV in T2 and L5 of both OI (+ 242% and + 218%, respectively) and Wt (T2: + 213%, L5: + 134%) in such a way that OI Scl-Ab showed significantly higher BV/TV than Wt Veh. (T2: + 151%, L5: + 70%). Trabecular bone sectional area (TBS) of T2 and L5 was not significantly different between OI Veh. and Wt Veh. Scl-Ab treatment significantly increased TBS of both OI (T2: + 132%, L5: + 96%) and Wt (T2: + 42%, L5: + 99%). At the end of the experiment, OI Scl-Ab had even higher TBS than Wt Veh. in T2 (+ 54%).

**TABLE 3:** Trabecular bone volume/total volume (BV/TV) and trabecular bone sectional area (TBS) in vertebral body of T2 and L5 measured in microradiographs of sagittal sections through the spine in male wild-type (Wt) and oim/oim (OI) mice after 9-week treatment with either Scl-Ab or vehicle (Veh.).

		Wt Veh.	Wt Scl-Ab	OI Veh.	OI Scl-Ab
		*n* = 10	*n* = 10	*n* = 10	*n* = 10
T2	BV/TV (%)	17.1 ± 2.8 c***	53.4 ± 12.6 b***	12.1 ± 5.7	42.8 ± 13.1 b***
	Gender difference (%)	−53.4**	+11.8	−6.0	−18.7
	TBS (mm^2^)	0.35 ± 0.08 c**	0.49 ± 0.04 b**	0.23 ± 0.09	0.53 ± 0.12 b***
	Gender difference (%)	+5.1	−22.4**	−24.9	+ 11.7
L5	BV/TV (%)	21.7 ± 2.4 c***	50.9 ± 9.3 b***	11.6 ± 5.7	36.9 ± 12.7 b***
	Gender difference (%)	+39.7***	+19.5**	−13.2	−4.1
	TBS (mm^2^)	1.22 ± 0.18	2.43 ± 0.34 b***	0.81 ± 0.39	1.59 ± 0.34 b***
	Gender difference (%)	+20.7*	+14.9**	+7.8	−8.4

The data from females used for gender differences (%) were obtained in the same experimental conditions, as described previously (Cardinal et al., 2020). Values are mean ± SD. b = Scl-Ab versus Veh., c = OI Scl-Ab versus Wt Veh. *p < 0.05, **p < 0.01, ***p < 0.001.

By comparison with females, male Wt Veh. had significantly smaller BV/TV in T2 and higher BV/TV and TBS in L5 at 14 weeks (Table 3). Scl-Ab treatment enhanced less TBS of T2 in male than in female Wt mice, whereas male Wt Scl-Ab had higher BV/TV and TBS in L5 than female Wt Scl-Ab. There was no difference in BV/TV and TBS between male and female OI groups whatever the treatment.

### Long-Bone Analysis

At 14 weeks, the tibias of OI Veh. mice were significantly shorter than those of Wt Veh (Table 4). Scl-Ab therapy did not change the length of Wt tibias, but was associated with a significant increase (+6%) in the tibia length of OI. At the end of the experiment, the length of the tibia remained significantly lower in OI Scl-Ab than in Wt Veh.

**TABLE 4:** Length, midshaft cross-sectional area (CSA), bone mineral density (BMD), polar stress–strain Index (SSI), cortical thickness (Ct.Th), and relative bone volume (BV/TV) of the tibia in male wild-type (Wt) and oim/oim (OI) mice after 9-week treatment with either Scl-Ab or vehicle (Veh.).

	Wt Veh.	Wt Scl-Ab	OI Veh.	OI Scl-Ab
	*n* = 10	*n* = 10	*n* = 10	*n* = 10
Length (mm)	18.2 ± 0.4 c**	18.3 ± 0.2	16.3 ± 0.9 a***	17.2 ± 0.6 b**
Gender difference (%)	+2.75***	+1**	−0.6	+2.3*
BMD (mg/cm^3^)	837.9 ± 60.8 c***	1010.4 ± 46.8 b***	840.57 ± 79.95	959 ± 40.81 b***
Gender difference (%)	−5.7	−4.7*	+ 11.9**	−0.9
SSI (mm^3^)	0.36 ± 0.08	0.55 ± 0.12 b***	0.22 ± 0.04 a**	0.31 ± 0.07
Gender difference (%)	+ 33.3***	+ 20*	+ 31.8**	+ 22.5**
CSA (mm^2^)	1.41 ± 0.22	1.87 ± 0.29 b***	1.01 ± 0.11 a***	1.21 ± 0.18
Gender difference (%)	+ 13.4*	+ 6.4	+ 17.8***	+ 5.7

	***n* = 8**	***n* = 8**	***n* = 8**	***n* = 8**

Ct.Th (mm)	0.22 ± 0.03	0.37 ± 0.02 b***	0.17 ± 0.02 a**	0.26 ± 0.02 b***
Gender difference (%)	−4.5	+ 8.1*	−10.5*	−3.8
BV/TV (%)	60.59 ± 4.07	75.20 ± 3.45 b***	53.48 ± 4.07 a**	65.34 ± 3.45 b***
Gender difference (%)	−5.6*	−0.1	−9.5*	−10.3***

Length was computed with X-ray, Ct.Th and BV/TV with microradiography, CSA, BMD, and SSI with pQCT. The data from females used for gender differences (%) were obtained in the same experimental conditions, as described previously (Cardinal et al., 2019). Values are mean ± SD. a = OI Veh. versus Wt Veh.; b = Scl-Ab versus Veh., c = OI Scl-Ab versus Wt Veh. *p < 0.05, **p < 0.01, ***p < 0.001.

In the absence of Scl-Ab treatment, BMD of the tibia was not significantly different between the OI and the Wt mice (Table 4). Scl-Ab therapy significantly increased the BMD in both OI and Wt mice (respectively, + 14%, + 21%). At the end of the study, the mean BMD of OI Scl-Ab was significantly higher than that of Wt Veh. (+ 15%). The polar SSI, computed with pQCT, was significantly lower in OI Veh. tibias than in Wt Veh. tibias (−58%). Scl-Ab enhanced SSI in both Wt and OI animals, but this difference was significant only in Wt ones (Wt Scl-Ab + 54%, OI Scl-Ab + 40%). After 9 weeks of therapy, there was no statistically significant difference in SSI between tibias of OI Scl-Ab and Wt Veh.

The midshaft Ct.Th, CSA, and bone volume/total volume (BV/TV) were significantly lower in OI Veh. than in Wt Veh. (respectively, −30, −39, −13%) (Table 4). Scl-Ab enhanced significantly Ct.Th in both groups (Wt Scl-Ab + 65%, OI Scl-Ab + 52%). A significantly positive effect of Scl-Ab on CSA was also observed in Wt Scl-Ab, but the increase in OI Scl-Ab was not significant (respectively, + 33%, + 19%). BV/TV was also significantly increased by therapy (Wt Scl-Ab + 24%, OI Scl-Ab + 22%). In OI Scl-Ab, Ct.Th, CSA, and BV/TV did not differ from those of Wt Veh. mice.

Male tibias were significantly longer than female ones in all groups except OI Veh (Table 4). BMD was lower in Wt males than females, but this difference was significant only in the Wt Scl-Ab, whereas it was significantly higher in male than female OI Veh. In all groups, SSI of the tibia was significantly higher in the males than in the corresponding females. Male versus female differences were observed for Ct.Th in Wt Scl-Ab (+8.1%) and in OI Veh. (−10.5%), for CSA in Wt Veh. (+13.4%) and in OI Veh. (+17.8%). BV/TV of the tibia was significantly lower in males than females except in the Wt Scl-Ab group.

**FIGURE 5:**
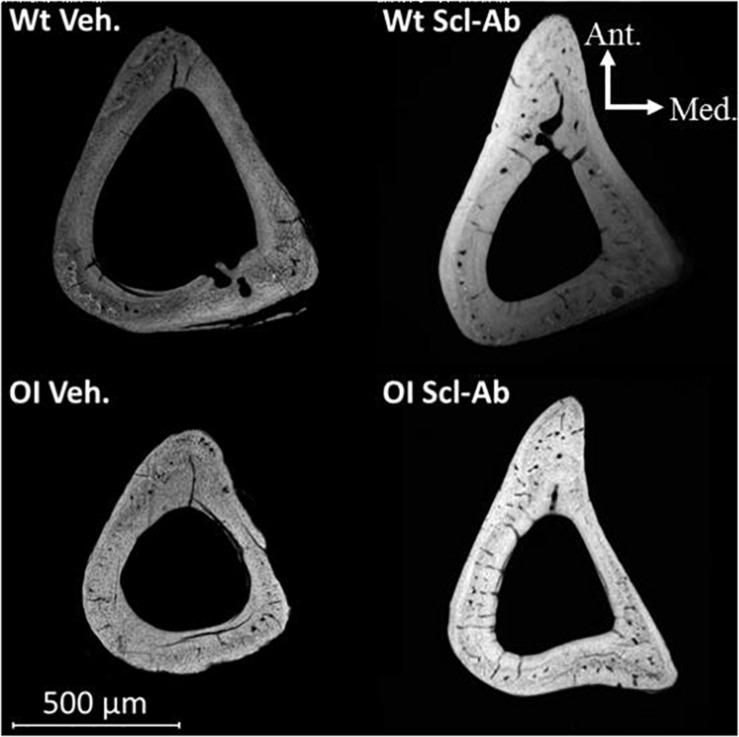
Microradiograph of a cross section through the tibial shaft of male mice illustrating differences in bone mass and geometry between OI Veh. and Wt Veh. mice, as well as the effect of Scl-Ab on these characteristics in both groups.

At microradiographic analysis of cross sections in tibial diaphysis, the shaft and the cortical bone appeared thinner in OI Veh. than Wt Veh (Figure 5). Both shaft diameter and Ct.Th appeared increased in mice treated with Scl-Ab as compared with Veh.-treated mice, whereas the medullary cavity appeared slightly narrower. Furthermore, numerous cavities were visible in cortical bone of OI Veh. and OI Scl-Ab.

**FIGURE 6:**
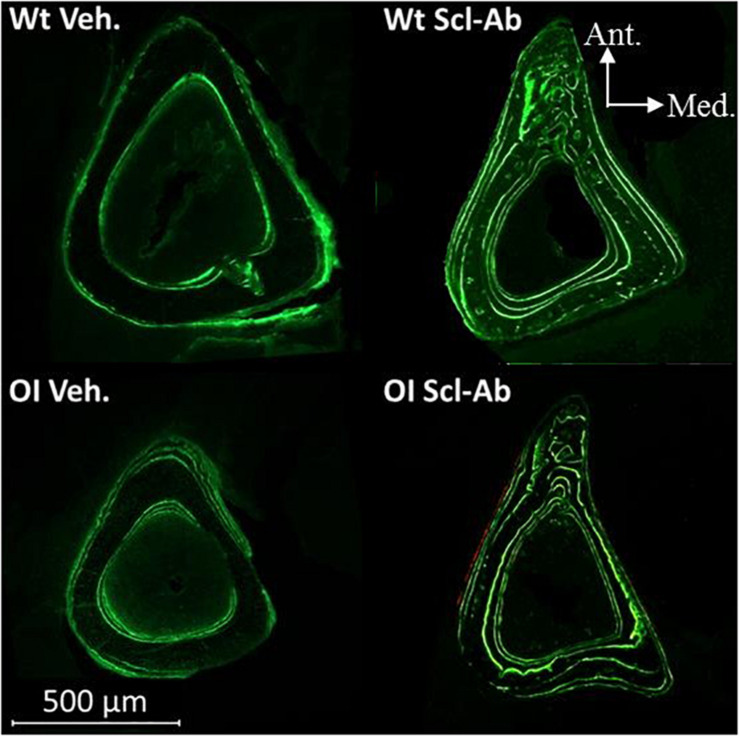
Fluorescence microscopy aspect of cross sections through the tibias of male Wt Veh., Wt Scl-Ab, OI Veh., and OI Scl-Ab mice. Green lines correspond to calcein injections at experimental days 1, 21, 42, and 63.

Under fluorescent microscopy, cross sections through tibial middiaphysis showed longer calcein labels on the endocortical and periosteal surfaces after Scl-Ab therapy than in vehicle-treated controls (Figure 6). The endocortical and periosteal bone surfaces located between green calcein labels appeared also thicker in Scl-Ab–treated OI mice than in vehicle-treated OI mice.

Endocortical MAR (E. MAR) was lower in tibias from OI Veh. than Wt Veh. mice (−42%), whereas the periosteal MAR (P. MAR) was not different (Table 5). Scl-Ab–treated animals showed significantly higher P. MAR (Wt Scl-Ab + 123%, OI Scl-Ab + 99%), E. MAR (Wt Scl-Ab + 49%, OI Scl-Ab + 65%), and P. BFR/BS (Wt Scl-Ab + 83%, OI Scl-Ab + 61%) than Veh. ones. At the end of therapy, P. MAR and P. BFR/BS were significantly higher in OI Scl-Ab–treated mice than in vehicle-treated Wt (respectively, + 92%, + 106%). Male Wt Veh. showed significantly higher E. MAR and E. BFR/BS than females (respectively, + 29.1%, + 34.5%; Table 5) whereas no difference was observed between male and female OI Veh. In male Wt Scl-Ab, both E. MAR and P. MAR were significantly higher than those in females, as well as P. BFR/BS and E. BFR/BS. These parameters were not significantly different between male and female OI Scl-Ab mice.

**TABLE 5:** Midshaft periosteal (P) and endocortical (E) mineral apposition rate (MAR) and bone formation rate per bone surface (BFR/BS, mm^3^/mm^2^ per year) of the tibia in male wild-type (Wt) and oim/oim (OI) mice after 9-week treatment with either Scl-Ab or vehicle (Veh.).

	Wt Veh.	Wt Scl-Ab	OI Veh.	OI Scl-Ab
	*n* = 6	*n* = 6	*n* = 6	*n* = 6
E. MAR (μm/d)	1.37 ± 0.38	2.05 ± 0.29 b**	0.8 ± 0.28 a**	1.32 ± 0.25 b*
Gender difference (%)	+ 29.1*	+ 39**	+ 27.5	+ 15.9
P. MAR (μm/d)	0.77 ± 0.24 c**	1.72 ± 0.25 b***	0.8 ± 0.3	1.59 ± 0.44 b**
Gender difference (%)	−16.8	+ 32.5**	+ 30	+ 19.4
P. BFR/BS	85.7 ± 23.2 c**	157 ± 21.8 b**	101.59 ± 37.57	164.44 ± 39.2 b*
Gender difference (%)	−18.3	+ 19.1*	+ 17.8	+ 9.4
E. BFR/BS	113.39 ± 33.09	118.87 ± 24.78	77.32 ± 24.59	97.72 ± 17.98
Gender difference (%)	+ 34.5*	+ 29.9*	+ 16.5	+ 14.1

The data from females used for gender differences (%) were obtained in the same experimental conditions, as described previously (Cardinal et al., 2019). Values are mean ± SD. a = OI Veh. versus Wt Veh.; b = Scl-Ab versus Veh., c = OI Scl-Ab versus Wt Veh. *p < 0.05, **p < 0.01, ***p < 0.001.

## Discussion

Bisphosphonates are the most commonly used treatment in OI patients. They reduce bone turnover and increase BMD, but do not consistently improve fracture incidence in pediatric controlled trials (Ward et al., 2011). Furthermore, BP might impair bone remodeling, decrease bone quality, and have toxic effects in mineralized tissue cell lines (Marini and Blissett, 2013). As anabolic agents strengthen bone and reduce fractures by stimulating bone formation (Sinder et al., 2014), increasing the amount of abnormal OI bone matrix was hypothesized to contribute to enhance OI bone strength and decrease the rate of fractures. These last years, blocking sclerostin (Scl) with an antibody (Scl-Ab) restored bone mass and strength in human postmenopausal osteoporosis (McClung et al., 2014; Recker et al., 2015) and in different animal models of postmenopausal (Li et al., 2009), age-related (Li et al., 2010) and disuse osteoporosis (Tian et al., 2011).

Recently, we tested Scl-Ab treatment in female oim/oim mice, which exhibit the main skeletal phenotype aspects of human OI (Cardinal et al., 2019, 2020). This treatment was shown to improve long-bone and vertebral morphometric, densitometric, and biomechanical parameters and to decrease fracture rate. In the present study, we assessed these parameters in male oim/oim and demonstrated some significant differences from those of female oim/oim, as well as distinct effects of Scl-Ab on male oim/oim.

Previous studies highlighted gender-related differences in bone morphometry and mechanical strength of oim/oim (Vanleene et al., 2012; Yao et al., 2013) and in their muscular and bone tissue parameters (Rao et al., 2008; Gentry et al., 2010), as well as in their response to therapeutic experiments (Boskey et al., 2015; Zimmerman et al., 2018). However, these studies contradict somewhat each other, which may be partially explained by differences in the age at sacrifice. Other studies did not separately analyze male and female oim/oim (Camacho et al., 1999; Bargman et al., 2012) or claimed no differences between both genders (Misof et al., 2005).

Gender-specific bone responses of oim/oim to treatment were demonstrated by Evans et al. for the first time after high doses of alendronate (Evans et al., 2003). Vanleene et al. (2011) reported a different reaction of male and female oim/oim mice to transplantation of human fetal bone stem/stromal cells. On the contrary, when testing soluble activin receptor type IIB-mFc (sActRIIB-mFc), a myostatin receptor, in +/G610C and oim/oim mice, Jeong et al. (2018) observed an anabolic effect on bone without gender correlation. Knowing that gender is a known determinant factor in bone strength and that Scl-Ab restored equally the BMD in osteoporotic men and women (Lindahl et al., 2014), we investigated the gender-related differences in clinimetric data and long-bone and vertebral parameters of oim/oim in order to highlight possible differences in response to Scl-Ab treatment. To our knowledge, no data are available about these differences in human with OI. Such gender-related analyses should be performed with other validated models of OI in order to take the variety of mutations into account when assessing gender specificities of the disease.

### Male OI Versus Wt

Male OI Veh. weighed less and had a shorter stature than the corresponding Wt mice during juvenile period (5–14 weeks). As shown in our data, at 14 weeks, the vertebral body of male OI Veh. was shorter than that of Wt Veh., along the whole spine, but this difference was significant in only a few thoracic vertebrae. These summed differences could explain the short stature of OI mice. The tibia was significantly shorter in OI Veh. than in Wt Veh. Stature and weight of types III and IV OI children were previously shown to be negatively affected, whereas gender affected only weight. Moreover, contrary to healthy teenagers, severe types of OI patients (III and IV) of both genders have no pubertal thrust (Barber et al., 2019).

Scl-Ab treatment did not change differences in stature and vertebrae length between oim/oim and Wt mice. In contrast, it appeared to have a positive impact on the tibias of OI Scl-Ab mice, although it did not allow reaching the same length as the Wt Veh. one. Aside from our long-bone length data, similar results were obtained using Scl-Ab in other strains of OI mice (Marenzana et al., 2011; Sinder et al., 2013; Roschger et al., 2014; Little et al., 2017). Indeed, no improvement in growth was observed using the antibody, suggesting that longitudinal growth modulation is less sensitive than periosteal growth after such therapy, which may explain the low impact of Scl-Ab on growth and body size.

Different bone mass and volumetric parameters (BMC, vertebral body CSA, and midshaft tibia CSA, Ct.Th, and BV/TV) were also smaller in OI Veh. than in Wt Veh. Osteoblastic activity was lower only at the endocortical surface of oim/oim long bones than in Wt. These results are corroborated by the studies cited just before. Interestingly, BMD, BV/TV, and trabecular bone sectional area (TBS) of vertebral body were not different between OI and Wt treated with vehicle. The treatment increased almost all these pQCT and histomorphometric data. Only CSA data did not change.

The improvement in bone mass parameters after Scl-Ab treatment can be attributed to rising osteoblastic activity, as observed in tibias of both Wt and OI groups. Indeed, Scl-Ab inhibits sclerostin, which is a potent inhibitor of the canonic Wnt pathway in osteoblasts. MAR and BFR/BS, which are histomorphometric indices of osteoblast activity, were improved in Wt as well as OI mice, particularly at the periosteal surface. Similar results were observed in cortical bone and mostly in trabecular bone of Col1a2 + /p.G610C mouse, which models human type IV OI, and in a hindlimb-immobilization rat model (Tian et al., 2011; Jacobsen et al., 2014).

The tibia of male oim/oim mice had also a lower SSI than the Wt one. Scl-Ab had a significant beneficial effect only on Wt tibia. However, the SSI increase in OI Scl-Ab allowed obtaining similar values as in the Wt Veh. This change could be responsible for reducing the fracture rate in male oim/oim.

### Male Versus Female OI

Contrary to Wt mice, clinimetric data from male OI were significantly inferior to those of females at 5 weeks, but these differences had disappeared at 14 weeks as male OI Veh. had similar stature, weight, and BMC as their female counterparts. At 14 weeks, T2 and L5 vertebral bodies tended to be longer in male Wt and OI than in female mice, this difference was significant in T2. Contrary to other observations (Evans et al., 2003), tibias of male oim/oim were not longer than those of female oim/oim in the present experiment. The Scl-Ab increased the tibia length of male oim/oim, which was not the case in the tibia of female OI Scl-Ab (Cardinal et al., 2019). The length of T2 vertebral body increased in male and female oim/oim after Scl-Ab administration, whereas the other mice groups did not show any change in long-bone and vertebral length with the treatment.

Tibia CSA, BMD, and SSI were higher in male oim/oim than in female. The same differences were found by Yao et al. (2013), who mentioned a higher tibia bone mass in male than in female mice, with significantly greater cortical bone density. Furthermore, the cortical porosity and thus the propagation of cracks are lower in males than in females, and the external diameter of long bones is indeed smaller in females than in males from 4 to 40 weeks of age in CB2-deficient mice, which show a markedly accelerated age-related bone loss (Bab, 2007). Yao et al. (2013) found similar trabecular data and BMD in vertebrae of both genders. However, in another study (Vanleene et al., 2012), female oim/oim showed a significantly higher BMD than male at 2 months. Our data showed the same trend for some thoracic vertebrae.

These pQCT and histomorphometric data evolved similarly in male and female Wt and OI after Scl-Ab treatment. However, male Wt showed the best response to Scl-Ab because their osteoblastic parameters outdid those of female mice. This difference was not significant in oim/oim mice. The Scl-Ab treatment induced a similar positive effect on bone mass and geometry in males and females of Wt and oim/oim groups. Furthermore, BMD of the tibia and all vertebral bodies was higher in oim/oim Scl-Ab than in Wt Veh. This impact of Scl-Ab on bone mass has also been observed in osteoporotic women and men (McClung et al., 2014; Recker et al., 2015). Therefore, it would be interesting to determine whether the effectiveness of treatment varies according to gender during teenhood in OI patients.

Tibias of male OI and Wt had a significantly higher SSI than female, which might be due to the fact that CSA was higher in males. Yao et al. had already reported better biomechanical properties of long bones in male than female oim/oim and Wt (Yao et al., 2013). Boskey et al. (2015) demonstrated higher cortical mineral-to-matrix ratio in male than female oim/oim. In absence of treatment, we observed a significantly higher fracture number in male than female oim/oim at 14 weeks. Therefore, regarding the phenotype aspects, male oim/oim seems to be a pertinent mouse model of human OI type III. After Scl-Ab treatment, the number of fractures was significantly lower in male than female oim/oim, suggesting a gender-related difference of efficiency of Scl-Ab.

In human, it is well established that sex steroids and growth hormone influence bone development (Arabi et al., 2004). Healthy teenager girls are known to have a higher BMD than boys. This difference could be attributed to the fact that young girls reach their peak bone mass earlier than boys during puberty, respectively, at the age of 16 years for healthy girls and 18 years for healthy boys (Boot et al., 2010). Kok et al. (2013) mentioned similar results for 17-year-old OI patients. Testing Scl-Ab in human OI patients should address possible interactions between sex steroids and growth hormone concentrations and the bone reactions.

## Conclusion

Our results, like other studies, showed differences in bone parameters between male and female oim/oim. In particular, males were frailer than females at 5 weeks and rather stronger at 14 weeks. The Scl-Ab treatment resulted in a similar positive effect on bone mass in both sexes. In the tibia, Scl-Ab stimulated periosteal bone formation in male oim/oim more than in females, leading to a better resistance to fracture. Our results provide justification for considering separately male and female oim/oim mice when testing any treatment, but also other mice models/mutations and, by extension, human patients suffering from OI.

## Data Availability Statement

The original contributions presented in the study are included in the article/Supplementary Material, further inquiries can be directed to the corresponding author/s.

## Ethics Statement

The animal study was reviewed and approved by the Comité d’Ethique pour l’Expérimentation Animale, Secteur des Sciences de la Santé, UCLouvain: 2014/UCL/MD/021.

## Author Contributions

J-PD, DM, and CB conceived and designed the study. MC, SL, and TR analyzed and interpreted the data. MO participated in data analysis. MC and AC wrote the manuscript. AC, DM, and CB revised and edited the manuscript. All authors contributed to the article and approved the submitted version.

## Conflict of Interest

MO is a former employee of Radius Inc. and a former employee and stockholder of Amgen Inc. The remaining authors declare that the research was conducted in the absence of any commercial or financial relationships that could be construed as a potential conflict of interest.

## Publisher’s Note

All claims expressed in this article are solely those of the authors and do not necessarily represent those of their affiliated organizations, or those of the publisher, the editors and the reviewers. Any product that may be evaluated in this article, or claim that may be made by its manufacturer, is not guaranteed or endorsed by the publisher.
