# Supplementary material for: Gender-Related Impact of Sclerostin Antibody on Bone in the Osteogenesis Imperfecta Mouse
Source: Front Genet. 2021 Aug 10;12:705505. doi: 10.3389/fgene.2021.705505 (PMC8383339; doi:10.3389/fgene.2021.705505)
Supplement: Supplementary file 1 [file Data_Sheet_1.pdf]

Supplementary Material – Annex

Highlights

Male vs Female oim/oim	Scl-Ab in male vs female oim/oim
- Weight, snout-sacrum length and bone mineral content were significantly lower in male than female oim/oim at 5w. No difference more at 14w.<br><br>- Long bone fractures significantly more numerous in male than female oim/oim at 14w.<br><br>- Cortical thickness and bone volume / total volume ratio of tibial diaphysis significantly lower in male than female oim/oim at 14w.<br><br>- No difference in bone volume / total volume ratio and trabecular bone sectional area of vertebral body between male and female oim/oim.	- No effect of Scl-Ab on weight and length gain in both male and female oim/oim, which remained significantly smaller and lighter than Wt at 14w.<br><br>- Number of fractures lower ($p < 0.05$) in male than female oim/oim after 9 w of Scl-Ab treatment.<br><br>- Scl-Ab increased periosteal mineral apposition rate in male oim/oim in such a way that tibial cortical thickness was not significantly different from female oim/oim at 14w.<br><br>- Bone mineral density and bone volume / total volume ratio of vertebral body increased in Wt and oim/oim with Scl-Ab, without significant difference between male and female at 14w.

Male versus female differences (%) in vertebral body bone mineral density (BMD), cross sectional area (CSA) and length of C3 to C7 according to their condition, Wt or OI, and treatment, Veh. or Scl-Ab. The data from females were obtained in the same experimental conditions, as described previously (16).

**Male vs female difference (%)**		**Wt Veh.**	**Wt Scl-Ab**	**OI Veh.**	**OI Scl-Ab**
C3	BMD	-14.9% *	-14.3% **	NS	NS
	CSA	NS	NS	NS	NS
	Length	NS	NS	NS	NS
C4	BMD	NS	NS	NS	NS
	CSA	NS	NS	NS	NS
	Length	NS	NS	NS	NS
C5	BMD	-15.3% ***	NS	NS	NS
	CSA	NS	NS	NS	NS
	Length	NS	+18.6% **	NS	NS
C6	BMD	-16.1% ***	NS	NS	NS
	CSA	NS	NS	NS	NS
	Length	NS	+19.6% **	NS	NS
C7	BMD	-16.0% ***	NS	NS	NS
	CSA	NS	NS	NS	NS
	Length	NS	+16.0% *	NS	NS

Values are mean, * = p <0.05; ** = p <0.01; *** = p <0.001. NS = not significant.

Male versus female differences (%) in vertebral body bone mineral density (BMD), cross sectional area (CSA) and length of T1 to T12 according to their condition, Wt or OI, and treatment, Veh. or Scl-Ab. The data from females were obtained in the same experimental conditions, as described previously (16).

**Male vs female difference (%)**		**Wt Veh.**	**Wt Scl-Ab**	**OI Veh.**	**OI Scl-Ab**
T1	BMD	-15.3% ***	-9.2% *	-14.8% **	NS
	CSA	NS	NS	NS	NS
	Length	NS	+14.8 *	NS	NS
T2	BMD	-15.2% ***	NS	NS	NS
	CSA	NS	NS	NS	NS
	Length	NS	NS	NS	NS
T3	BMD	-14.3% ***	NS	NS	NS
	CSA	NS	+28.4% *	NS	NS
	Length	NS	+13.8% *	NS	NS
T4	BMD	-12.9% **	NS	NS	NS
	CSA	NS	NS	NS	NS
	Length	NS	+17.3% ***	NS	NS
T5	BMD	-12.9% *	NS	NS	NS
	CSA	NS	NS	NS	NS
	Length	NS	NS	NS	NS
T6	BMD	NS	NS	NS	NS
	CSA	NS	NS	NS	NS
	Length	NS	+12.0% *	NS	+10.0% *
T7	BMD	NS	NS	-16.1% **	NS
	CSA	NS	NS	NS	+18.3% *
	Length	NS	NS	NS	NS
T8	BMD	-13.2% **	NS	-14.7% **	NS
	CSA	NS	NS	NS	NS
	Length	NS	+11.2% **	NS	NS
T9	BMD	-12.7% *	NS	NS	NS
	CSA	NS	NS	NS	NS
	Length	NS	+12.7% **	NS	NS
T10	BMD	-13.0% *	NS	NS	NS
	CSA	NS	NS	NS	NS
	Length	NS	+13.5% ***	NS	NS
T11	BMD	NS	NS	NS	NS
	CSA	NS	NS	NS	NS
	Length	NS	+15.0% ***	NS	+9.1% *
T12	BMD	NS	NS	NS	NS
	CSA	NS	NS	NS	NS
	Length	NS	+9.8% *	NS	NS
Values are mean, * = p <0.05; ** = p <0.01; *** = p <0.001. NS = not significant.					

Male versus female differences (%) in vertebral body bone mineral density (BMD), cross sectional area (CSA) and length of L2 to L6 and of S1 to S4 according to their condition, Wt or OI, and treatment, Veh. or Scl-Ab. The data from females were obtained in the same experimental conditions, as described previously (16).

**Male vs female difference (%)**		**Wt Veh.**	**Wt Scl-Ab**	**OI Veh.**	**OI Scl-Ab**
L2	BMD	NS	NS	NS	NS
	CSA	NS	NS	NS	NS
	Length	NS	NS	NS	NS
L3	BMD	-23.1% ***	NS	NS	NS
	CSA	NS	NS	NS	NS
	Length	NS	NS	NS	NS
L4	BMD	NS	NS	NS	NS
	CSA	+ 12.3% *	NS	NS	NS
	Length	NS	NS	NS	NS
L5	BMD	NS	NS	NS	NS
	CSA	NS	+9.4% *	+18.8% *	NS
	Length	NS	NS	NS	NS
L6	BMD	NS	NS	NS	NS
	CSA	+14.7% ***	+15.4% ***	+20.9% **	NS
	Length	NS	NS	NS	NS
S1	BMD	NS	NS	NS	NS
	CSA	NS	+13.9% ***	NS	+12.1% ***
	Length	X	X	X	X
S2	BMD	NS	NS	NS	NS
	CSA	+13.9% **	NS	NS	+17.3% ***
	Length	X	X	X	X
S3	BMD	NS	-11.4% *	NS	NS
	CSA	+15.9% ***	NS	NS	+10.1% **
	Length	X	X	X	X
S4	BMD	NS	-12.8% **	NS	NS
	CSA	+13.8% **	+7.9% *	NS	+10.7% **
	Length	+6.0% *	+6.2% *	NS	+5.6% *

Values are mean, * = p <0.05; ** = p <0.01; *** = p <0.001. NS = not significant.
